# The Role of Statins in the Management of Patients Undergoing Coronary Artery Bypass Grafting

**DOI:** 10.3390/diseases6040102

**Published:** 2018-11-11

**Authors:** Dimitrios Siskos, Konstantinos Tziomalos

**Affiliations:** 1 Department of Cardiothoracic Surgery, Heart Center, University Hospital Cologne, 50937 Cologne, Germany; dimitrios.siskos@uk-koeln.de; 2 First Propedeutic Department of Internal Medicine, Medical School, Aristotle University of Thessaloniki, AHEPA Hospital, 54636 Thessaloniki, Greece

**Keywords:** statins, coronary artery bypass grafting, pleiotropic actions, atrial fibrillation, stroke, acute kidney injury

## Abstract

Each year, a large number of patients undergo coronary artery bypass grafting surgery (CABG) worldwide. Accumulating evidence suggests that the preoperative administration of statins might be useful in preventing adverse events after CABG. In the present review, we discuss the role of statins in the perioperative management of patients undergoing CABG. Preoperative administration of statins in these patients substantially reduces the risk of postoperative atrial fibrillation and shortens hospital and intensive care unit (ICU) stay. Atorvastatin appears to be more effective, particularly when administered at high doses. Given these benefits and the safety of statins, their administration should be considered in patients undergoing CABG, even though the statins do not appear to affect the incidence of cardiovascular events and overall mortality perioperatively.

## 1. Introduction

Each year, a large number of patients undergo coronary artery bypass grafting surgery (CABG) worldwide [1]. Despite improved surgical techniques, the advances in technology used in the operating room (new cardiopulmonary bypass machines and cannulas, more precise sensors and monitoring), improved cardiac anesthesiologic management, and the more experience gathered by surgeons over the years, CABG is still associated with a considerable risk for adverse events, including death [1]. As a result, lowering postoperative morbidity is not only an intraoperative challenge, but also depends on preoperative management. In this context, it is worthy to look back over the publications from years 2000–2007, accumulating evidence that suggests that the preoperative administration of statins might be useful in preventing adverse events after CABG [2,3,4].

In the present review, we discuss the role of statins in the perioperative management of patients undergoing CABG.

## 2. Effects of Statins on Atrial Fibrillation after CABG

In the Atorvastatin for reduction of myocardial Dysrhythmia after cardiac surgery (ARMYDA-3 study), a randomized, placebo-controlled study (*n* = 200), treatment with atorvastatin (40 mg/day for seven days) before cardiac surgery (not only isolated CABG) reduced the incidence of postoperative atrial fibrillation by 61% [5]. In a meta-analysis of 3 randomized controlled trials (RCTs) and 10 observational studies (*n* = 17,643), statin treatment before CABG reduced the risk for any postoperative atrial fibrillation by 36% and for new-onset atrial fibrillation by 34% [6]. In a more recent meta-analysis of 12 RCTs in 2980 patients, statin treatment before CABG reduced the incidence of postoperative atrial fibrillation by 58% [7]. Notably, atorvastatin reduced the risk by 65%, whereas rosuvastatin was not protective [7]. Rosuvastatin also did not reduce the incidence of post-CABG atrial fibrillation in the Statin Therapy In Cardiac Surgery (STICS) trial, a large RCT (*n* = 1922) [8]. The reason for the different outcome between atorvastatin and rosuvastatin is mainly unknown. In addition, it should be mentioned that only two small studies evaluated the effects of the administration of pravastatin and simvastatin on the risk of atrial fibrillation in patients undergoing CABG (*n* = 43 and 44, respectively) [7]. Even though these studies showed no effect of these statins on the incidence of atrial fibrillation, more data are clearly needed to clarify whether these statins might also prevent this complication. In addition, there are no studies that evaluated the effects of other statins (fluvastatin, pitavastatin) on the incidence of atrial fibrillation in patients undergoing CABG [7].

Regarding the association between statin dose and the risk of atrial fibrillation, an early retrospective study showed that a high-dose statin treatment reduces the risk for atrial fibrillation after CABG more than an intermediate-dose treatment, whereas a low-dose treatment has no protective effect [9]. In contrast, in a duration- and dose-response meta-analysis of eight RCTs (*n* = 774), there was no association between statin dose and risk reduction (*p* = 0.47) [10]. Nevertheless, it should be emphasized that both the lipid-lowering and the pleiotropic effects of statins (e.g., anti-inflammatory, antioxidant, and antithrombotic actions) as well as the reduction in cardiovascular risk with these agents are clearly dose-dependent [11]. Therefore, it is possible that this meta-analysis included a small number of patients and did not have the statistical power to show an association between statin dose and the incidence of atrial fibrillation. On the other hand, in the former meta-analysis, a longer duration of preoperative statin treatment was associated with a greater reduction in the risk of postoperative atrial fibrillation (3% reduction per day of statin treatment, *p* = 0.008) [10]. 

The physiological mechanism(s) underpinning the preventive effect of statin against postoperative atrial fibrillation are still unknown. The pleiotropic effects of statins, including anti-inflammatory, antioxidant, and antithrombotic actions as well as inhibition of neurohormonal activation, appear to play a role [11,12].

## 3. Effects of Statins on Hospital and Intensive Care Unit (ICU) Stay after CABG

Several studies showed that preoperative statin therapy shortens hospital stay by approximately 0.5 days [7,10,13,14,15]. Preoperative statin therapy also reduces postoperative ICU stay, but this reduction is more modest (by 3–4 h) [10,13]. It is possible that the reduction in the incidence of atrial fibrillation and its associated complications, including embolic stroke and hemodynamic instability, contribute to the shortening of hospitalization in patients who receive statins prior to CABG [7,10,13,14,15].

## 4. Effects of Statins on Renal Failure, Myocardial Infarction, Stroke, and Mortality after CABG

Postoperative acute kidney injury is a frequent complication in patients undergoing CABG [12,13,16,17,18]. Observational studies suggested that pretreatment with statins reduces the risk of postoperative renal failure and the need for hemodialysis in this population [19,20,21]. In contrast, an RCT showed that atorvastatin does not affect the incidence of acute kidney injury after CABG [22] whereas in the STICS trial, the rate of postoperative acute kidney injury was higher in patients who received rosuvastatin prior to CABG compared with patients who received a placebo [8]. Several systematic reviews and meta-analyses concluded that treatment with statins before CABG does not affect the risk of acute kidney injury [12,13,18].

Preoperative treatment with statins does not appear to reduce the risk for postoperative myocardial infarction (MI) [13,17]. However, a meta-analysis of eight studies (n = 8676) showed that patients who receive a loading dose of statin prior to CABG have a lower risk for non-fatal and fatal MI as well as a lower risk for graft restenosis and repeat CABG than patients who receive a regular dose [23]. In a prospective observational study, patients who received high-intensity statin treatment (i.e., expected to reduce low-density lipoprotein cholesterol (LDL-C) levels by >45%) had a lower risk for cardiovascular events than patients who received moderate-intensity statin treatment (i.e., expected to reduce LDL-C levels by <45%) [24].

The incidence of postoperative ischemic stroke also does not appear to be affected by the administration of statins before CABG [13,16,17]. However, a single-center study suggested that the combination of statins with beta blockers reduces the risk for ischemic stroke after CABG [25]. Clearly, more studies are needed to confirm this promising finding.

An early retrospective cohort study suggested that pretreatment with statins is associated with reduced postoperative 30-day all-cause mortality after CABG [17]. However, several more recent RCTs and meta-analyses did not identify any survival benefit with statin treatment in these patients [13,15,16].

## 5. Conclusions

The preoperative administration of statins in patients undergoing CABG substantially reduces the risk of postoperative atrial fibrillation and shortens hospital and ICU stay. Atorvastatin appears to be more effective, particularly when administered at high doses. Given these benefits and the safety of statins, their administration should be considered in patients undergoing CABG, even though the statins do not appear to affect the incidence of cardiovascular events and overall mortality perioperatively. More studies are needed to clarify the mechanisms underpinning these beneficial effects of statins and to define the optimal compound, dose, and duration of treatment.
